# Antitumor and anti-cachectic effects of shark liver oil and fish oil: comparison between independent or associative chronic supplementation in Walker 256 tumor-bearing rats

**DOI:** 10.1186/1476-511X-12-146

**Published:** 2013-10-16

**Authors:** Fabíola Iagher, Sérgio Ricardo de Brito Belo, Wanessa Mazanek Souza, Juliana Rehlander Nunes, Katya Naliwaiko, Guilherme Lanzi Sassaki, Sandro José Ribeiro Bonatto, Heloísa Helena Paro de Oliveira, Gleisson Alisson Pereira Brito, Carina de Lima, Marcelo Kryczyk, Carine Ferreira de Souza, Jovani Antonio Steffani, Everson Araújo Nunes, Luiz Cláudio Fernandes

**Affiliations:** 1 Area of Biological and Health Sciences, West University of Santa Catarina, Joaçaba, Brazil; 2 Department of Physiology, Biological Science Building, Cell Metabolism Laboratory, Federal University of Parana, Curitiba, Brazil; 3 Department of Cell Biology, Biological Science Building, Federal University of Parana, Curitiba, Brazil; 4 Department of Biochemistry and Molecular Biology, Biological Science Building, Federal University of Parana, Curitiba, Brazil; 5 Department of Physiological Sciences, Biological Sciences Center, Laboratory of Investigation in Chronic Diseases, Federal University of Santa Catarina, Florianópolis, Brazil

**Keywords:** Cachexia, Fish oils, Walker 256, Lipid peroxidation, Apoptosis, Cell proliferation, Rats, Wistar

## Abstract

**Background:**

Shark liver oil (SLOil) and fish oil (FOil), which are respectively rich in alkylglycerols (AKGs) and n-3 polyunsaturated fatty acids (PUFAs), are able to reduce the growth of some tumors and the burden of cachexia. It is known that FOil is able to reduce proliferation rate and increase apoptotic cells and lipid peroxidation of tumor cells efficiently. However, there are few reports revealing the influence of SLOil on these parameters. In the current study, effects of FOil chronic supplementation on tumor growth and cachexia were taken as reference to compare the results obtained with SLOil supplementation. Also, we evaluated if the association of SLOil and FOil was able to promote additive effects.

**Methods:**

Weanling male Wistar rats were divided into 4 groups: fed regular chow (C), supplemented (1 g/kg body weight) with SLOil (CSLO), FOil (CFO) and both (CSLO + FO). After 8 weeks half of each group was inoculated with Walker 256 cells originating new groups (W, WSLO, WFO and WSLO + FO). Biochemical parameters of cachexia, tumor weight, hydroperoxide content, proliferation rate and percentage of apoptotic tumor cells were analysed. Fatty acids and AKG composition of tumor and oils were obtained by high performance liquid chromatography and gas chromatography – mass spectrometry, respectively. Statistical analysis was performed by unpaired t-test and one-way ANOVA followed by a post hoc Tukey test.

**Results:**

Fourteen days after inoculation, SLOil was able to restore cachexia parameters to control levels, similarly to FOil. WSLO rats presented significantly lower tumor weight (40%), greater tumor cell apoptosis (~3-fold), decreased tumor cell proliferation (35%), and higher tumor content of lipid hydroperoxides (40%) than observed in W rats, but FOil showed more potent effects. Supplementation with SLOil + FOil did not promote additive effects. Additionally, chromatographic results suggested a potential incorporation competition between the n-3 fatty acids and the AKGs in the tumor cells’ membranes.

**Conclusions:**

SLOil is another marine source of lipids with similar FOil anti-cachectic capacity. Furthermore, despite being less potent than FOil, SLOil presented significant *in vivo* antitumor effects. These results suggest that the chronic supplementation with SLOil may be adjuvant of the anti-cancer therapy.

## Background

Cancer susceptibility is determined by genetic factors; however, environmental factors seem to influence which subjects genetically susceptible will be affected. In this context, nutrition has been aroused as a main component in such relation [1]. Nutrition has a central role in this feature, because it can be applied as a preventive tool or as a component of the anti-cancer therapy when the disease is already installed. Among several other nutrients, lipids receive a special attention in such line of thinking. Phospholipids and other lipids, incorporated into cell membranes or other compartments, can have their molecular composition altered depending on the lipid nutrition profile of the subject. This fact justifies the importance of studies investigating the repercussions of different lipid nutritional supply on cancer progress. One of the most common manifestations of cancer is the development of cachexia syndrome, a chronic wasting condition responsible for the loss of both adipose and skeletal muscle tissues. At least 20% of the deaths among cancer patients are due to cachexia [2]. This syndrome involves immune-metabolic pathways, and so far, the mechanisms by which it happens remain not fully understood.

Western countries have a diet rich in saturated and n-6 polyunsaturated fatty acids (PUFAs). Such a diet is commonly low in n-3 PUFAs and vitamins C and E, which have been associated with the development of some cancers. Fish oil (FOil) is a source of long chain n-3 PUFAs, such as eicosapentaenoic acid (EPA) and docosahexaenoic acid (DHA). These n-3 PUFAs have been shown to decrease the risk for several cancers [3,4], the tumor growth [5-7], and cancer cachexia [8,9] in both animal models [6,10,11] and clinical trials [3,9,12]. The mechanisms by which n-3 PUFAs cause such effects are not fully understood. Noteworthy, the participation of increased lipid peroxidation in tumor tissue [6,7], reduction of proinflammatory cytokines and chemical mediators that induce cell proliferation [7,13,14], and promotion of tumor cell apoptosis [7,11,15,16] have been reported.

Another marine compound that contains n-3 PUFAs is shark liver oil (SLOil). Besides the n-3 PUFAs, SLOil also presents alkylglycerols (AKGs) in its composition. These compounds are constituted by glycerol linked to the hydrocarbon tail in the *sn-1* position by ether bonds. AKGs can be associated to fatty acids by ester bonds in the *sn-2* and *sn-3* positions, constituting alkyldiacylglycerol molecules [17]. AKGs represent about 20% of the shark liver oil lipid composition [18]. These ether lipids are found in hematopoietic organs of mammals, especially in the bone marrow and in human breast milk. SLOil seems to be an immune system enhancer [19,20], and this effect is attributed in part to AKGs. The first clinical studies using SLOil supplementation were for the treatment of leukemia and also as a complementary agent administered to uterine cervix cancer patients submitted to X-ray therapy. In such approaches, SLOil supplementation was able to avoid leukopenia and thrombocytopenia usually caused by radiation [21]. There are *in vivo* studies showing that SLOil [17] as well as isolated AKGs are able to reduce tumor growth [18]. Recently, our group showed that chronic SLOil supplementation was able to reduce tumor growth and cachexia [22,23], but the action mechanisms involved in this reduction are not fully understood and need to be investigated. Moreover, no study has investigated the effects of a long-term supplementation with SLOil plus FOil on tumor and cachexia development. Whereas that FOil is the main source of n-3 PUFAs and SLOil is the main source of AKGs, the association of both oils could play an additive effect.

Thus, the aim of this work was to investigate some mechanisms involved in tumor growth arrest in Walker 256 tumor-bearing rats chronically supplemented with SLOil, to evaluate the effects on biochemical parameters of cachexia, and to compare the results with those obtained from animals supplemented with FOil. Furthermore, it was investigated if the supplementation with SLOil plus FOil was able to exert additive effects. To accomplish that, we determined the tumor weight, *ex vivo* tumor cell proliferation rate, lipid peroxidation and apoptosis in tumor tissue. Body mass, liver glycogen content, serum concentrations of triacylglycerol, glucose, and lactate have also been evaluated.

## Results

FA composition of tumor tissues was modified by the supplementation protocol (Table 1). In FOil supplemented animals (WFO and WSLO + FO), the n-3 PUFAs level in tumor tissues was ~2-fold higher than that in SLOil supplemented animals and ~4-fold higher when compared to the W group (p < 0.05). SLOil was able to decrease the arachidonic acid (AA) concentration in tumor tissues in relation to the W group (p < 0.05); however, FOil (WFO and WSLO + FO) promoted an even more significant reduction (p < 0.05).

**Table 1:** Fatty acid profiles of SLOil, FOil, regular chow, and tumor tissue

**Fatty acids (g/100g of total fatty acids)**	**SLOil**	**FOil**	**Regular chow**	**Tumor tissue**			
				**W**	**WSLO**	**WFO**	**WSLO + FO**
Lauric (12:0)	1.3 ± 0.0	3.2 ± 0.3	1.1 ± 0.0	0.4 ± 0.1	0.5 ± 0.1	0.4 ± 0.1	0.4 ± 0.1
Miristic (14:0)	5.9 ± 0.1	8.7 ± 2.6	-	1.1 ± 0.4	0.7 ± 0.0	1.0 ± 0.2	1.0 ± 0.2
Palmitic (16:0)	34.2 ± 3.7	24.8 ± 2.9	22.3 ± 0.3	24.1 ± 0.9	26.1 ± 2.5	22.8 ± 1.5	26.5 ± 1.5
Palmitoleic (16:1n-7)	4.7 ± 0.4	5.5 ± 0.4	-	2.7 ± 0.1	1.4 ± 0.5	1.7 ± 0.1	1.6 ± 0.2
Stearic (18:0)	2.2 ± 0.1	2.8 ± 0.1	1.9 ± 0.0	15.7 ± 1.9	15.1 ± 0.9	8.0 ± 0.9	9.1 ± 1.4
Oleic (18:1n-9)	29.6 ± 3.1	11.8 ± 0.7	20.4 ± 0.9	24.8 ± 2.6	24.1 ± 3.0	24.8 ± 0.9	25.7 ± 0.9
Linoleic (18:2n-6)	2.1 ± 0.2	2.0 ± 0.5	50.6 ± 3.2	18.5 ± 1.3	22.9 ± 3.1	21.8 ± 2.3	22.6 ± 2.5
α-Linolenic (18:3n-3)	0.3 ± 0.0	0.8 ± 0.0	3.9 ± 0.2	0.5 ± 0.0	0.2 ± 0.0	1.0 ± 0.07	0.9 ± 0.2
Arachidonic (20:4n-6)	1.1 ± 0.1	0.9 ± 0.0	0.1 ± 0.0	16.9 ± 1.3	8.4 ± 2.1^a^	3.5 ± 0.8^a^	3.9 ± 1.4^a^
EPA (20:5n-3)	5.2 ± 0.5	21.3 ± 1.1	-	0.3 ± 0.1	0.6 ± 0.1	1.2 ± 0.2^a^	0.8 ± 0.2^a^
DHA (22:6n-3)	11.4 ± 1.0	17.9 ± 1.4	-	1.0 ± 0.0	2.0 ± 0.2	4.2 ± 0.7^ab^	4.2 ± 0.5^ab^

Tumor tissue from Walker 256 tumor-bearing rats fed regular chow (W), supplemented with SLOil (WSLO), FOil (WFO) and both (WSLO + FO), determined by HPLC (high performance liquid chromatography). Data are presented as mean ± SEM of 3 independent measurements (HPLC) of 3 rats per group (n = 9). ^a^p < 0.05 compared to W; ^b^p < 0.05 compared to WSLO.

SLOil was composed by 80% of the main AKG, octadecenylglycerol, hexadecylglycerol and octadecylglycerol (Table 2). The tumor tissue of animals supplemented only with SLOil (WSLO) showed AKG concentration significantly higher than that in the other groups (p < 0.05) (Table 3). The main AKGs found in SLOil were ~1.6-fold higher in WSLO tumors than in W tumors and ~3-fold in relation to groups supplemented with FOil (WFO and WSLO + FO).

**Table 2:** Alkylglycerol composition of SLOil determined by GC-MS

**Alkylglycerols**	**% of total alkylglycerols**
Octadecenylglycerol (C18:1)	53.0
Hexadecylglycerol (C16:0)	18.1
Octadecylglycerol (C18:0)	8.9
Others	20.0

GC-MS indicates gas chromatography – mass spectrometry.

**Table 3:** Alkylglycerol composition of tumor tissue

**Alkylglycerols (ng/mg of tumor tissue)**	**Groups**			
	**W**	**WSLO**	**WFO**	**WSLO + FO**
Hexadecylglycerol (C16:0)+				
Octadecylglycerol (C18:0)+	4.6 ± 0.7	7.6 ± 0.3^a^	2.8 ± 0.2	2.2 ± 0.3
Octadecenylglycerol (C18:1)				

Tumor tissue from Walker 256 tumor-bearing rats fed regular chow (W), supplemented with SLOil (WSLO), FOil (WFO) or both (WSLO + FO), determined by GC-MS (gas chromatography – mass spectrometry). Data are presented as mean ± SEM of 3 independent measurements (GC-MS) of 3 rats per group (n = 9). ^a^p < 0.05 compared to other groups.

Non-tumor-bearing animals gained around 18 g in 2 weeks (Table 4), and oil supplementation did not cause any further gain. On the other hand, tumor presence caused a significant loss of body weight in the W animals (~19 g). SLOil (WSLO) prevented wasting as well as FOil (WFO) and both (WSLO + FO), which were similar to the control ones (CSLO, CFO and CSLO + FO, respectively). The tumor weight in the W animals was 18.4 ± 1.0 g. The supplementation with SLOil induced a significant reduction in the tumor weight, which was 10.9 ± 1.1 g. FOil supplementation caused a more potent effect, and the tumor weight was 7.3 ± 0.6 g. Both oil supplementations did not cause any further effect on the tumor weight, similar to FOil alone (8.2 ± 0.6 g).

**Table 4:** Body, tumor and carcass weight of non-tumor bearing rats (prefix C), and Walker 256 tumor-bearing rats (prefix W)

**Group**	**Body weight before tumor inoculation (g)**	**Body weight 14 days after inoculation (g)**	**Tumor weight on 14th day (g)**	**Carcass on 14th day (g)**	**Weight change (g)**
C	322.3 ± 17.5	338.4 ± 16.8	-	-	+16.2 ± 2.0
CSLO	317.4 ± 13.5	336.5 ± 12.2	-	-	+17.9 ± 3.9
CFO	316.8 ± 7.8	334.4 ± 7.2	-	-	+17.7 ± 2.1
CSLO + FO	331.5 ± 7.0	351.1 ± 7.7	-	-	+19.6 ± 2.3
W	321.3 ± 17.3	319.0 ± 11.9	18.4 ± 1.0	301.5 ± 11.6	−18.5 ± 1.4^b^
WSLO	320.1 ± 6.7	348.8 ± 7.9	10.9 ± 1.1^a^	337.9 ± 7.1	+12.9 ± 2.5^a^
WFO	317.3 ± 11.6	338.3 ± 8.8	7.3 ± 0.6^ac^	331.1 ± 8.7	+15.5 ± 0.9^a^
WSLO + FO	327.0 ± 9.6	349.4 ± 12.6	8.2 ± 0.6^a^	340.9 ± 13.2^a^	+18.5 ± 1.9^a^

Rats fed regular chow (C and W), supplemented with SLOil (CSLO and WSLO), FOil (CFO and WFO) and both (CSLO + FO and WSLO + FO). Data are presented as mean ± SEM of 4 independent experiments of 5 rats per group (n = 20). ^a^p < 0.05 compared to W; ^b^p < 0.01 compared to C; ^c^p < 0.01 compared to WSLO.

Among non-tumor-bearing animals (C, CSLO, CFO, and CSLO + FO groups), glycemia, triacylglycerolemia, lactatemia, and liver glycogen content were not different (p > 0.05) (Table 5). W animals presented reduced glycemia, hypertriacylglycerolemia, hyperlactatemia, and reduced liver glycogen content characterizing cachexia state. SLOil (WSLO) and FOil (WFO) supplementation were able to keep glycemia and lactatemia levels significantly different from those of the W group (p < 0.05). The association of both oils (WSLO + FO) did not cause any further modification (p > 0.05 *vs*. WSLO and WFO).

**Table 5:** Serum concentrations of glucose, triacylglycerol (TAG) and lactate, and liver glycogen content of non-tumor (prefix C), and Walker 256 tumor-bearing rats (prefix W)

** Groups**	**Glucose (mg/dL)**	**TAG (mg/dL)**	**Lactate (mmol/L)**	**Liver Glycogen (μmol/g tissue) (w/w)**
C	97.4 ± 3.2	67.4 ± 3.4	1.28 ± 0.1	137.5 ± 5.9
CSLO	96.8 ± 2.2	71.8 ± 3.8	1.27 ± 0.1	137.7 ± 5.5
CFO	98.7 ± 3.3	63.2 ± 6.1	1.39 ± 0.1	156.8 ± 7.2
CSLO + FO	94.9 ± 2.3	66.9 ± 3.2	1.27 ± 0.1	148.5 ± 8.4
W	78.7 ± 1.5^a^	113.4 ± 9.4^a^	2.26 ± 0.1^a^	94.5 ± 9.8^a^
WSLO	87.9 ± 2.5^b^	92.3 ± 4.4	1.47 ± 0.1^b^	124.7 ± 2.3^b^
WFO	99.9 ± 3.9^b^	78.9 ± 4.7^b^	1.5 ± 0.1^b^	125.2 ± 5.6^ab^
WSLO + FO	94.4 ± 2.3^b^	83.8 ± 4.6^b^	1.43 ± 0.1^b^	125.4 ± 3.5^ab^

Rats fed regular chow (C and W), supplemented with SLOil (CSLO and WSLO), FOil (CFO and WFO) and both (CSLO + FO and WSLO + FO). Data are presented as mean ± SEM of 4 independent experiments of 5 rats per group (n = 20). ^a^p < 0.01 compared to respective group without tumor; ^b^p < 0.05 compared to W group.

**Figure 1:**
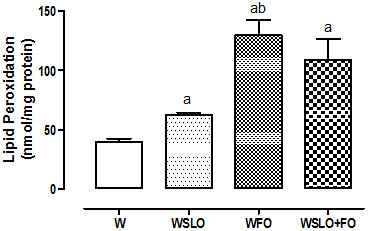
**Lipid hydroperoxide concentration in tumor tissue.** Tumors from animals fed regular chow (W), supplemented with SLOil (WSLO), FOil (WFO) and both (WSLO + FO). Values are mean ± SEM of 3 independent assays; 8 (W) (n = 24), 6 (WSLO) (n = 18), 6 (WFO) (n = 18) and 5 (WSLO + FO) (n = 15) rats. ^a^p < 0.001 compared to W; ^b^p < 0.05 compared to WSLO.

Tumor samples from rats fed with SLOil (WSLO) showed concentration of lipid hydroperoxides around 1.6-fold greater than those fed with regular chow (W). Animals supplemented with FOil (WFO) and those supplemented with both oils (WSLO + FO) presented lipid peroxidation ~3-fold higher than tumor samples from W rats (p < 0.001). WFO tumor samples had a lipid peroxidation by ~2-fold higher than that from WSLO group (p < 0.05) (Figure 1).

**Figure 2:**
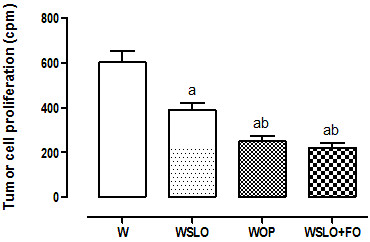
***Ex vivo *proliferation rate (counts/min) of 256 Walker tumor cells.** Tumors from animals fed regular chow (W), and supplemented with SLOil (WSLO), FOil (WFO) and both (WSLO + FO). Data are presented as mean ± SEM of 3 independent assays of 10 rats per group (n = 30). ^a^p < 0.001 compared to W, ^b^p < 0.05 compared to WSLO.

Supplementation with SLOil (WSLO) promoted reduction by ~1.5-fold in the proliferation capacity when compared to the group fed regular chow (W) (p < 0.001) (Figure 2). Animals from WFO and WSLO + FO groups showed similar reduction of proliferation capacity, i.e., ~2.5-fold vs. W (p < 0.001) and ~1.5-fold vs. WSLO (p < 0.05).

The analysis of tumor cells by flow cytometry (Table 6) revealed that SLOil supplementation (WSLO) was able to increase by ~3-fold the percentage of apoptotic cells, and reduce by ~2.4-fold the necrotic cells and by ~1.7-fold the quantity of viable cells in comparison to animals fed regular chow (W) (p < 0.001). When FOil was applied alone (WFO) and in association with SLOil (WSLO + FO), the results obtained were similar, with slight increase of apoptotic cells percentage in relation to animals supplemented only with SLOil (p < 0.05).

**Table 6:** Percentage of apoptotic, necrotic and viable cells of 256 Walker tumors

**Groups**	**Apoptotic cells (%)**	**Necrotic cells (%)**	**Viable cells (%)**
**W**	19.8 ± 2.3	30.6 ± 1.1	49.6 ± 2.7
**WSLO**	56.9 ± 1.0^a^	12.8 ± 1.2^a^	28.9 ± 1.5^a^
**WFO**	68.5 ± 2.9^ab^	7.5 ± 0.9^ab^	23.7 ± 1.0^a^
**WSLO + FO**	64.8 ± 0.6^ab^	13.5 ± 0.5^a^	21.6 ± 0.7^ab^

Values determined by FACS (fluorescence-activated cell sorting), from animals fed regular regular chow (W), and supplemented with SLOil (WSLO), FOil (WFO), and both (WSLO + FO). Data are presented as mean ± SEM of 4 independent experiments of 5 rats per group (n = 20). ^a^p < 0,05 compared to W; ^b^p < 0,05 compared to WSLO.

## Discussion

The role of diet in cancer development has been extensively studied in the last years [1,4-7,12,15]. It has been advocated that 30–40% of cancers may be prevented by appropriated diets, physical activity, and maintenance of body weight [24]. The 256 Walker tumor is a good tool to study cachexia. All the features presented in the W group are commonly seen in cachectic individual [6,25].

The group supplemented with FOil only has been taken in this work as a reference point to compare with SLOil effects. The reduction of about 60% in the tumor weight of the WFO group has been reported in previous studies that used the same experimental model [6,7]. FOil supplementation was able to maintain body weight and metabolic parameters in the tumor-bearing rats (WFO) to similar values when compared to the non-tumor-bearing group (CFO). These results are in agreement with those obtained by other studies with animal models [6,10,25]. Supplementation with SLOil also reduced tumor growth by ~40%, as showed in a previous study of our group [23]. Although the tumor-bearing rats (SLOil supplemented) have tumor size ~30% bigger than FOil supplemented rats, they completely reversed the cachexia state. Some studies have suggested that cachexia in Walker 256 tumor-bearing rats is directly connected to tumor size [10,25]. However, as we have reported in a previous study [6], here we show that such association is not always present. More important is that FOil and SLOil have similar efficient anti-cachectic properties in this model.

The capsules of the SLOil and FOil used in the present study contain EPA and DHA (Table 1). However, in addition to n-3 PUFAs, the SLOil capsules used here are rich in AKGs (Table 2), which are not found in FOil and regular rodent chow. Pédrono et al. [18] showed that AKGs purified from SLOil and the whole oil supplemented orally to animals reduced tumor growth similarly, indicating that anti-tumor activity of SLOil is probably due to AKG. AKG can be inserted in the cell membranes as ether phospholipids and serve as substrates for the formation of second messengers with ether linkage, which can interfere with the activity of enzymes involved in the control of cell proliferation and differentiation, such as protein kinase C (PKC) [20,26,27]. The anti-tumor effect of SLOil has been associated to the interaction of 1-O-alkyl-2-acylglycerol, an analogue of diacylglycerol (DAG), with PKC [28]. In addition, studies with cultured cells suggest that ether phospholipids, such as n-3 fatty acids, can be accumulated in lipid rafts and act on cell-signaling by affecting the protein composition of these microdomains [29].

SLOil contains n-3 PUFAs and AKGs in its composition, and it cannot be discarded that tumor and cachexia reduction in WSLO animals may be the result of the combined action of both compounds. It has been known that n-3 PUFAs have the ability to decrease production of tumor factors involved in the promotion of cachexia [2], and this fact may have contributed to the reduction of cachexia in animals supplemented with FOil (WFO) and SLOil (WSLO). However, SLOil has only half the concentration of n-3 PUFAs than FOil, so AKG also may exert active effect upon cachexia. Therefore, the mechanisms related to the effects of these ether lipids need to be further investigated.

The results obtained to EPA and DHA concentrations in the WSLO and WFO tumor tissues (~2.6% and ~5.4%, respectively; Table 1) are in agreement with n-3 PUFA content in SLOil and FOil capsules (~17% and ~39%, respectively). The WSLO tumor tissue showed half the percentage of AA in relation to W group, while WFO showed about 4-fold less AA than W group. Elevated concentrations of AA in tumor tissue are related to high prostaglandin E_2_ (PGE_2_) production by tumor, and, consequently, enhanced tumor development [7].

N-3 PUFAs are very susceptible to peroxidation, which explains the higher concentration of lipid hydroperoxides (~2-fold) found in tumors from the WFO group than those found in WSLO tumors (Figure 1). Peroxidation products are able to inhibit tumor growth by increasing apoptosis, contributing to antiproliferative effect of these compounds [30,31]. Indeed, results obtained by FACS showed that independent supplementation with SLOil and FOil promoted increase in apoptosis in relation to group W (Table 6). However, SLOil promoted slightly lower tumor cell apoptosis than FOil, even though half the concentration of n-3 PUFAs has been found in tumor tissue. A small difference also can be observed in the percentage of viable cells between the WSLO and WFO groups. Thus, it can be suggested that part of the pro-apoptotic effect may be attributed to AKGs from SLOil. Indeed, the concentration of AKGs in the WSLO tumor tissue has been around 3-fold greater than that found in WFO (Table 2). The proliferation capacity of tumor cells from the WSLO group was significantly reduced in relation to the W group (~1.5-fold), but the WFO group presented even bigger reductions (~2.4-fold; almost 50% lower than WSLO) (Figure 2). Although FOil has been more effective in reducing tumor cell proliferation, SLOil chronic ingestion showed important capacity to control tumor growth directly. The indirect anti-tumor action of SLOil by the stimulation of the immune system has not been studied here, but recently, our group found that the long-life exposure to SLOil was able to increase the nitrite production by the peritoneal macrophages. The nitrite production is indicative of NO production, and NO could contribute to the reduction of tumor growth in WSLO animals [22].

Contrary to our expectations, the association of SLOil and FOil (WSLO + FO group) did not cause additive effects on tumor growth reduction as well as in the cachexia parameters (Tables 4 and 5). Lipid hydroperoxide content in tumor tissues from WSLO + FO animals was not significantly different from those observed in WFO animals (Figure 1). In the same way, tumor cell proliferation capacity and percentage of apoptotic cells were similar in both groups (Figure 2 and Table 6, respectively). These results suggest that FOil is the main factor, and SLOil did not promote any further effect. Indeed, tumor tissue from the WSLO + FO group showed similar proportion of n-3 PUFAs, EPA, and DHA (Table 1) when compared to WFO and approximately twice the concentration found in WSLO tumors. Interestingly, the concentration of AKGs in the WFO and WSLO + FO groups was also similar and about 3-fold lower than that found in the WSLO group (Table 3). These results suggest that the incorporation of AKGs in tumor cells is reduced when there is another supply of fat in the diet. We suggest that when FOil, rich in n-3 PUFAs, and SLOil, rich in AKG, are applied together, n-3 PUFAs are preferentially incorporated into the tumor tissue, and the incorporation of AKG is significant only when SLOil is offered alone. This competition for incorporation into the lipid bilayer may help to explain why the effects on tumor growth, lipid peroxidation, proliferation capacity of tumor cell, apoptosis, and cachexia parameters observed in the WFO and WSLO + FO groups were identical.

## Conclusions

Here we show that independent chronic ingestion of SLOil is able to reduce Walker 256 tumor growth, and this effect is linked to increased lipid peroxidation, increased apoptosis, and reduced tumor cell proliferative capacity. Although FOil supplementation has been more effective to reduce tumor growth, SLOil and FOil showed equivalent anti-cachectic properties. Association of SLOil and FOil did not cause additive effect on tumor growth or cachexia and produced results similar to those found in animals supplemented with FOil alone, probably because of competition between n-3 PUFA and AKG for incorporation in the tumor cell membrane.

## Methods

### Study design

Procedures involving animals were approved by the Committee of Animal Welfare of the Federal University of Paraná. Weanling male Wistar rats (age, 21 days) were maintained under controlled temperature (23°C) and humidity in 12-h light/12-h dark cycle and randomized into 4 dietary groups. The control group (C) received a regular chow (protein content, 230 g/kg; fiber, 60 g/kg; fats, 40 g/kg; carbohydrates, 660 g/kg; vitamins and minerals, 10 g/kg; Nuvital CR-1; Curitiba, PR, Brazil). Three groups received, in addition to regular chow, daily fat supplementation (1 g/kg body weight) as follows: SLOil (CSLO), FOil (CFO), and SLOil + FOil (CSLO + FO, 1 g/kg body weight of each oil). After 8 weeks, 50% of the rats were inoculated subcutaneously in the right flank with 1 mL of a sterile suspension of 3 × 10^7^ Walker tumor cells that were obtained from an ascitic tumor-bearing rat. Walker 256 tumor-bearing groups were identified by the prefix W (W, WSLO, WFO, and WSLO + FO). Supplementation continued to be made for additional 2 weeks after tumor inoculation. Body weight was monitored every 2 days. Fourteen days after tumor inoculation, animals were killed by decapitation without anesthesia. The tumors were removed, weighed, and the samples were reserved for assays. Blood was collected into 15 mL tubes and allowed to clot for 30 min at room temperature. Serum was prepared by centrifugation and used for the measurement of lactate, glucose, and triacylglycerol concentrations. Liver pieces were excised and frozen to posterior glycogen content detection.

### Chemicals, oils, and enzymes

Chemicals and enzymes used in this study were obtained from Sigma Chemical Co. (St. Louis, MO, USA). Biochemical kits were obtained from BioLiquid® (Laborclin; Pinhais, PR, Brazil). FOil from Herbarium® and SLOil from Ecomer® were kindly donated by the Herbarium Foundation (Curitiba, PR, Brazil) and the Naturalis Alimentos Naturais Ltda (São Paulo, SP, Brazil), respectively.

### Biochemical parameters of cachexia

Serum lactate (mmol/L), glucose (mg/dL), and triacylglycerol (mg/dL) were analyzed immediately after the animals were killed. Liver was frozen in liquid nitrogen to posterior glycogen measurement. For the lactate assay, serum (0.5 mL) was added to 0.1 mL of perchloric acid (25%) and left for 10 min at 4°C followed by centrifugation at 3,000 *g* for 5 min. The supernatant was collected and neutralized with Tris/KOH (2 M/0.5 M), and the concentration of lactate was enzimatically determined as previously described [32] at 340 nm. Serum glucose and triacylglycerol measurements were performed with enzimatically colorimetric commercial BioLiquid® kits and quantified by measuring the absorbance at 505 nm and 540 nm, respectively. For the glycogen assay, accurately weighed pieces of liver (0.07 g) were put into 0.5 mL of KOH aqueous solution (1 mol/L) and left for 20 min at 70°C for tissue digestion. After that, a digested sample (0.1 mL) was added to 0.5 mL triethanolamine buffer containing amyloglucosidase and incubated for 2 h at room temperature. After centrifugation (800 *g* for 5 min), the supernatant (0.2 mL) was added to 1 mL of glucose assay buffer as previously described [33]. Glycogen was quantified by measuring the absorbance at 340 nm, and the calculated results were expressed as μmol/g of tissue (w/w). Data are presented as mean ± SEM of 4 independent measurements, 5 rats per group (n = 20).

### Determination of fatty acids (FAs) by using HPLC and AKGs by using GC-MS

The lipids were extracted [34] from standard commercial chow, SLOil, FOil, and tumor tissue (3 independent measurements, 3 rats per group, n = 9) and saponified by using 2 mL of an alkaline methanol solution (1 mol NaOH/L in 90% methanol) at 37°C for 2 h in a shaking water bath. Then, the alkaline solution was acidified to pH 3.0 with HCl solution (1 mol/L). FAs were then extracted 3 times with 2 mL hexane. After the extraction procedure and saponification [35,36], the FAs were derivatized with 4-bromomethyl-7 methoxycoumarim [37], and the analysis was performed on a Varian model LC-10A liquid chromatograph. The samples were placed on a C8 column (25 cm × 4.6 mm i.d.; 5 μm of particles) with a flow rate of 1 mL/min of acetonitrile/water (77:23, v/v) and a fluorescence detector (325 nm excitation and 395 nm emission). The standard mixture of FA was obtained from Sigma Chemical Co. (St. Louis, MO, USA). The elution sequence and limit of detection were determined. The minimum limit of quantification of the FAs ranged from 1–10 ng. One curve of calibration for each standard, determining the coefficients of correlation and regression, was obtained.

The AKG profile was determined by using GC-MS (gas chromatography–mass spectrometry) as follows: Total lipids were extracted from SLOil capsules and tumor tissue using chloroform-methanol (2:1 v/v) [34]. Then, the content was hydrolyzed and kept under 100°C for 2 h. The samples were dried out under nitrogen gas and added 0.2 mL of ethanoyl ethanoate and 0.2 mL of pyridine followed by 30 min of incubation at 100°C [38]. AKGs were separated on a GC-MS Saturn 2000R by using a CP-Sil-5 CB Chrompack® column (30 m × 0.25 mm). The results are presented as ng of AKG per mg of tumor tissue. An external standard galactitol, 48 ng/μL by injection was used for this assay. SLOil was composed of 50 mg of AKG/250 mg of oil.

### Tumor tissue lipid hydroperoxides

The products of peroxidation were measured using the method previously described [39]. Tumor tissue (0.2 mg) was homogenized in methanol (1 mL) and centrifuged, and 90 μL of the supernatant was added to the reaction tubes containing 10 μL of methanol or 10 μL of triphenylphosphine (10 mM). Thereafter, was added 0.9 mL of reaction solution (100 μM xylenol orange, 250 μM Fe^+2^, 25 mM H_2_SO_4_, and 4 mM butylated hydroxytoluene in 90% (v/v) methanol) and incubated for 30 min at room temperature prior to measurement at 560 nm. The concentration of lipid hydroperoxides was calculated by subtracting the absorbance of tryphenylphosphine samples from respective methanol samples, and values were expressed as nmol of lipid hydroperoxides/mg of protein. The protein content of the tumor tissue homogenates was measured by using the Bradford method [40], using bovine serum albumin (BSA) as the standard. Data are presented as mean ± SEM of 3 independent assays; 8 (W) (n = 24), 6 (WSLO) (n = 18), 6 (WFO) (n = 18) and 5 (WSLO + FO) (n = 15) rats.

### *Ex vivo* Walker 256 tumor cell proliferation

The whole tumor was removed and chopped with a scalpel. The cell suspension was obtained by filtration through funnel and gauze. Red blood cells were discharged with a solution containing NH_4_CL (15.5 mM). Tumor cells were prepared by centrifugation at 290 *g* for 7 min and were resuspended in RPMI 1640 medium. Tumor cells were cultured for 24 h at 37°C in an air/CO_2_ (19:1) atmosphere in 96-well microtiter-culture plates at a density of 1 × 10^5^ cells/well in RPMI 1640 medium enriched with 10% fetal calf serum containing 100 U/mL penicillin and 100 μg/mL streptomycin (Gibco; Grand Island, NY) and 0.05 μCi of [2-^14^C]-thymidine. The cells were harvested onto glass fiber disks (Filtermats; COX Scientific Ltd., Kettering, UK) and washed using a Titertek Cell Harvester (Skattron, Lier, Norway). Radioactive thymidine incorporation into DNA was determined by liquid scintillation counting in a Beckman LS 6000IC scintillation counter [33]. Results are presented as counts per minute (cpm)/10^5^ cells, of 3 independent assays, 10 rats per group (n = 30).

### Tumor cell flow cytometry analysis

The percentage of tumor cells undergoing apoptosis and necrosis was evaluated by using annexin V-FITC (fluorescein isothiocyanate; Produced by Institute of Biomedical Sciences, University of São Paulo, Brazil) and 7-AAD (7-amino-actinomycin D; BD Biosciences Pharmingen), respectively. Tumor cells (10^6^), isolated from tumors after tumor extraction, were stained with annexin V-FITC for 15 min and with 7-AAD for 5 min and then analyzed using flow cytometry (FACS – fluorescence-activated cell sorting). The fluorescence was analyzed using flow cytometer FACScalibur (Becton Dickinson; San Jose, CA). The tumor cell population was primarily selected from the forward-scattered light (FSC) vs. side-scattered light (SSC) dot plot in linear mode. Then, a FL1 vs. FL3 dot plot in log mode was constructed to quantify cells labeled with annexin V-FITC (FL1, low right quadrant) and cells labeled with 7-AAD (FL3, up left quadrant). Cells that were located in the low left quadrant (not labeled cells) were considered viable. The cells positive for annexin V-FITC were considered to be in an early stage of apoptosis. Data are presented as mean ± SEM of 4 independent measurements of 5 rats per group (n = 20).

### Statistical analysis

Data are presented as mean ± standard error of mean (SEM). Statistical analysis were performed by unpaired t-test and one-way analysis of variance (ANOVA) followed by a post hoc Tukey test, when diet or tumor was used as a factor. Data were analyzed using GraphPad prism software (version 5.0; GraphPad Software; San Diego, California). *P < 0.05* was taken to indicate statistical significance.

## Abbreviations

SLOil: Shark liver oil; AKG: Alkylglycerol; FOil: Fish oil; PUFA: Polyunsaturated fatty acid; EPA: Eicosapentaenoic acid; DHA: Docosahexaenoic acid; FA: Fatty acid; GC-MS: Gas chromatography – mass spectrometry; BSA: Bovine serum albumin; SDS-PAGE: Sodium dodecyl sulphate – polyacrylamide gel electrophoresis; 7-AAD: 7-amino-actinomycin; FITC: Fluorescein isothiocyanate; FACS: Fluorescence-activated cell sorting; FSC: Forward-scattered light; SSC: Side-scattered light; AA: Arachidonic acid; PKC: Protein kinase C; DAG: Diacylglycerol; PGE2: Prostaglandin E2.

## Competing interests

The authors declare that they have no competing interests.

## Authors’ contributions

FI wrote the manuscript. SRBB, WMS, JRN, KN, GLS, SJRB, HHPO, GAPB, CL, MK, CFS and JAS conducted data collection and analysis. EAN and LCF were involved on the review and edition of the manuscript. All authors made critical comments during study design and preparation of manuscript. All authors read and approved the final manuscript.
